# Correction: Durán-Ferrer et al. Clinical, Virological and Immunological Responses After Experimental Infection with African Horse Sickness Virus Serotype 9 in Immunologically Naïve and Vaccinated Horses. *Viruses* 2022, *14*, 1545

**DOI:** 10.3390/v18090964

**Published:** 2026-09-02

**Authors:** Manuel Durán-Ferrer, Rubén Villalba, Paloma Fernández-Pacheco, Cristina Tena-Tomás, Miguel-Ángel Jiménez-Clavero, José-Antonio Bouzada, María-José Ruano, Jovita Fernández-Pinero, Marisa Arias, Javier Castillo-Olivares, Montserrat Agüero

**Affiliations:** 1 Laboratorio Central de Veterinaria (LCV), Ministry of Agriculture, Fisheries and Food, Ctra. M-106, pk 1,4, 28110 Algete, Spain; mduran@mapa.es (M.D.-F.); rvillalba@mapa.es (R.V.); jbouzada@mapa.es (J.-A.B.); mruanor@mapa.es (M.-J.R.); 2 Centro de Investigación en Sanidad Animal (CISA), Instituto Nacional de Investigación y Tecnología Agraria y Alimentaria (INIA-CSIC), Ctra. M-106, pk 8,1, 28130 Valdeolmos, Spain; pacheco@inia.es (P.F.-P.); majimenez@inia.es (M.-Á.J.-C.); fpinero@inia.csic.es (J.F.-P.); arias@inia.es (M.A.); 3 Tecnologías y Servicios Agrarios, S.A, (TRAGSATEC), 28006 Madrid, Spain; at_algete9@mapa.es; 4 CIBER of Epidemiology and Public Health (CIBERESP), 28029 Madrid, Spain; 5 Laboratory of Viral Zoonotics, Department of Veterinary Medicine, University of Cambridge, Cambridge CB3 0ES, UK; fjc37@cam.ac.uk

**Error in Table 2.**

In the original publication [1], there was a mistake in Table 2. Serotype-specific rRT-PCR primers and probes targeting VP2 AHSV genes specific for each serotype, as published. The mistake lies in the nucleotide sequences of some primers and/or probes, specifically Serotype 2 (AHS-2F), Serotype 3 (AHS-3F and AHS-3R), Serotype 7 (AHS-7F, AHS-7R and AHS-7P) and Serotype 9 (AHS-9R and AHS-9P).

The corrected Table 2. Serotype-specific rRT-PCR primers and probes targeting VP2 AHSV genes specific for each serotype appears below. The authors state that the scientific conclusions are unaffected. This correction was approved by the Academic Editor. The original publication has also been updated.

## Figures and Tables

**Table 2:** Serotype-specific rRT-PCR primers and probes targeting VP2 AHSV genes specific for each serotype.

AHSV	Primers (F/R) and Probe	Sequence 5′-3′
Serotype 1	AHS-1F	GCAAGCGCTGGCACTTG
	AHS-1R	TTCGAACTCATTCCTTACATCAACA
	AHS1P	FAM-AATGTCTTAGATCGTCAACT-MGB
Serotype 2	AHS-2F	CGGAAACTYTGTATTGCCAAA
	AHS-2R	TTGTCRTCCTGATCAACCCTAA
	AHS-2P	Cy5-TGAAGGTGCTTACCCGATCTTTCCACA-BBQ
Serotype 3	AHS-3F	AATTATTACAGCGGAGAATGCAGTT
	AHS-3R	GGTTATGAGTGGGGTGCGA
	AHS-3P	FAM-AGAGTTGAGGTTGCGGGA-MGB
Serotype 4	AHS-4F	TGAGGTGGAACACGAYATGTC
	AHS-4R	GATATGCCCCCTCACAYCTGA
	AHS-4P	VIC-TATCGGRATTTATGTACAATGAG-MGB
Serotype 5	AHS-5F	GAAGAGACAGGCGATTCAAATGA
	AHS-5R	AAAGCCACCCTTTTTGGTACAAA
	AHS-5P	NED -TGTTGARATGCTGAGGC-MGB
Serotype 6	AHS-6F	AGCCAGGGCTTCTTTGCA
	AHS-6R	CTCATGTTCAACCCACTGTACATTAA
	AHS-6P	VIC-GTCATCACCGTAAGCG-MGB
Serotype 7	AHS-7F	AGCCAGGGCTTCTTTGCA
	AHS-7R	CTCATGTTCAACCCACTGTACATTAA
	AHS-7P	VIC-GTCATCACCGTAAGCG-MGB
Serotype 8	AHS-8F	GAAATTATCAGCGGACTGACTAAGAA
	AHS-8R	AAACATCTACCTTTTGCGAATCTTG
	AHS-8P	NED-ACGTGATTCTTTTCCC-MGB
Serotype 9	AHS-9F	TACTGTGTCGGTGAGGGATTTT
	AHS-9R	GCCACGACCGGATATGA
	AHS-9P	FAM-AAACAAACGAAATGTGAA-MGB

**Table 2:** Serotype-specific rRT-PCR primers and probes targeting VP2 AHSV genes specific for each serotype.

AHSV	Primers (F/R) and Probe	Sequence 5′-3′
Serotype 1	AHS-1F	GCAAGCGCTGGCACTTG
	AHS-1R	TTCGAACTCATTCCTTACATCAACA
	AHS1P	FAM-AATGTCTTAGATCGTCAACT-MGB
Serotype 2	AHS-2F	CGGAAACYATGTATTGCCAA
	AHS-2R	TTGTCRTCCTGATCAACCCTAA
	AHS-2P	Cy5-TGAAGGTGCTTACCCGATCTTTCCACA-BBQ
Serotype 3	AHS-3F	AATTATTACAGCGGARAATGCAGTT
	AHS-3R	TCGCACCCCACTCATAACC
	AHS-3P	FAM-AGAGTTGAGGTTGCGGGA-MGB
Serotype 4	AHS-4F	TGAGGTGGAACACGAYATGTC
	AHS-4R	GATATGCCCCCTCACAYCTGA
	AHS-4P	VIC-TATCGGRATTTATGTACAATGAG-MGB
Serotype 5	AHS-5F	GAAGAGACAGGCGATTCAAATGA
	AHS-5R	AAAGCCACCCTTTTTGGTACAAA
	AHS-5P	NED -TGTTGARATGCTGAGGC-MGB
Serotype 6	AHS-6F	AGCCAGGGCTTCTTTGCA
	AHS-6R	CTCATGTTCAACCCACTGTACATTAA
	AHS-6P	VIC-GTCATCACCGTAAGCG-MGB
Serotype 7	AHS-7F	GAGTGGGGCGCAACAAATC
	AHS-7R	CCGGATATGCACCCTCACAT
	AHS-7P	VIC-CGATATCTGAGTTCATGTATGAA-MGB
Serotype 8	AHS-8F	GAAATTATCAGCGGACTGACTAAGAA
	AHS-8R	AAACATCTACCTTTTGCGAATCTTG
	AHS-8P	NED-ACGTGATTCTTTTCCC-MGB
Serotype 9	AHS-9F	TACTGTGTCGGTGAGGGATTTT
	AHS-9R	GGCACGACCGGATATGA
	AHS-9P	FAM-TTCACATTTCGTTTGTTT-MGB

## References

[1] Durán-Ferrer M., Villalba R., Fernández-Pacheco P., Tena-Tomás C., Jiménez-Clavero M.-Á., Bouzada J.-A., Ruano M.-J., Fernández-Pinero J., Arias M., Castillo-Olivares J. (2022). Clinical, Virological and Immunological Responses after Experimental Infection with African Horse Sickness Virus Serotype 9 in Immunologically Naïve and Vaccinated Horses. Viruses.

